# Military Miscellany, Comprehending a History of the Recruiting of the Army, Military Punishments, &c.

**Published:** 1846-07

**Authors:** 


MILITARY MISCELLANY, COMPREHENDING A HISTORY OF THE RECRUITING OF THE ARMY, MILITARY PUNISHMENTS, &c. By *Henry Marshall*, F.R.S.E., Deputy Inspector-General of Army Hospitals, &c. Octavo, pp. 375. London, 1846. Murray.

IN a previous article in our present number, we have alluded to the great and most desirable reform that has, of late years, been effected in the treatment of the insane. Thanks be to God, men's eyes are now opened to the folly as well as the sin of punishing the worst of human misfortunes as a crime, and of attempting to subdue or correct the diseases of the mind by the infliction of suffering upon the body. Language like this may be considered as putting a too charitable construction on the motives of those who could act so; and truly we must confess that, in the majority of instances, it was cruel selfishness rather than want of knowledge, wilful insensibility rather than prejudice or error, in short, the evil passions of man's heart much more than the ignorance of man's head, that were the real causes of those terrible and most revolting enormities that used to be—would that they had utterly ceased to exist in the present day!—perpetrated within the walls of lunatic asylums. How humiliating a reflection it is that human beings, when left to themselves, unawed by the disgrace of detection or the fear of punishment, should treat their fellow-creatures worse than the very beasts beneath their feet! The vilest of mankind would blush to chain up a helpless brute in a dark damp cell, and there to leave it to wallow in its own mire, without clothing or sufficient food, but with no lack of blows, often and mercilessly inflicted. Yet such was actually the treatment that was too frequently the portion of him that is fashioned in his Creator's image, and made instinct with immortality. The faculties of the poor maniac's mind may indeed be unhinged and perverted, but it would seem to have been forgotten that still the mind is there; and, for ought that we know, the Almighty himself may condescend to hold intercourse with that very spirit, which man, in his impiety, supposes to be wholly eclipsed. It is only, we believe, by taking this high view of the subject, and by contemplating the relationship that subsists between all members of the human family to each other, and to Him who hath made and who knoweth all things, that we can ever hope to obtain a safe and unerring guide in our efforts to correct many of the worst evils of our social system. The science of political ethics has too often set all principles of justice, not to talk of mercy, at defiance. There is no want of instances to prove the truth of this assertion. Take, for example, the history of the criminal code in this or in any other nation of the earth. What, it may be fairly asked, has been the great defect in all legislative enactments upon this most important subject? Is it not the case that the only object and aim have uniformly been to deter by fear, on the one hand, and inflict condign punishment upon the offender on the other? Have the laws ever sought to prevent the commission of crime by duly instructing the people in the knowledge of their duties, and by encouraging them in their exercise? or have they, while they awarded pains and penalties for their infraction, simultaneously endeavoured to correct the evil principles or enlighten the ignorance that led to that infraction? Hitherto little, very little, has been done, or even sought to be done, in either of these ways. A criminal is detected, apprehended, thrust into the company of those more hardened in vice than himself, tried, convicted, and punished—let us suppose by imprisonment. What is the ordinary result? he comes out more depraved and corrupted than before; none will employ or trust him; he cannot starve; he is forced almost by necessity to the the commission of a new crime; and thus things go on from bad to worse, until, perhaps, his life is at length forfeited to the laws, or he is driven a disgraced outcast to a penal land. Now something very similar to all this has been, in one respect at least, the condition of our soldiers and sailors; and the plan of treatment, that has been pursued towards them, has been, —until within the last 30 years or so—founded upon equally enlightened and humane principles. A man was found guilty of some (it might be very petty) offence, and forthwith he was ordered to have 100 lashes. The same or a similar offence was repeated; 300 was the number now assigned. A third time, the effect of 500 was now tried; but still without permanent or decided amendment of the offender. Well, what then? Did officers begin to perceive the inefficacy of their invariable panacea? Oh, no; the dose, it was said, had not been sufficient; and forthwith 1000, 1200, nay 1500 lashes were ordered, and inflicted too, upon the wretched criminal. On reading of such cases, the question naturally occurs to one's mind, was this corporal punishment at all likely to correct any vicious habits; to make the drunkard sober, the thief honest, or the coward brave? What does the testimony of military men themselves say upon this head? All, without exception, who have written or given public evidence upon the subject—whether they be abolitionists, or not—agree on this one point, that flogging never made a good out of a bad soldier. Nor can we wonder at it. A human creature, when treated like a brute, becomes gradually hardened and indifferent as to himself; he loses all self-respect; and *therefore* he is utterly regardless of what others think or say of him. This is a law of man's moral nature; who can gainsay its truth? You may torture his body, you may load him with fetters, and shut him out from the face of day; you may crush his very soul with injuries; but you cannot, by mere force, extract the poison-fang of vice that is rooted in his breast. How, then, is this to be effected? We shall answer the question by a short narrative. A man had been over and over again flogged; but this repeated punishment seemed to have really no effect at all in deterring him from disobedience. At length, "the commanding officer," says our author, "observing that, notwithstanding all his vices, he had some very valuable qualifications, resolved to try another mode than whipping. It was not long before he had an opportunity of putting his scheme into execution; for the next fault, instead of being punished, to the fellow's great surprise, he appointed him Sergeant! This opened his eyes, he applied himself diligently to his duty, and became as remarkably sober and good as he had been the contrary before."

Sir Neil Douglas, the gallant Colonel of the 79th, understood well the art of managing his men; for he tells us :—

"To this mode of punishment (flogging) I have been a great enemy, having found from long experience that it is hopeless to expect any good results from its infliction, either as a *warning to good men to avoid evil courses, or as a punishment to bad ones* for the actual commission of crime. I therefore gave much attention to the subject, and came to the conclusion, after mature reflection, that under the existing system of military law of England, my only chance of abating it, was by stimulating men's minds by holding out other great advantages for good and regular behaviour, and thus making it their interest to conduct themselves with propriety.' For this purpose Sir Neil established a regimental order of merit in the corps he commanded, the 79th Regiment of Highlanders, 'which,' he says, 'tended more than any measure I ever knew or heard of, to encourage good conduct, and to repress vice and immorality of every description.' Sir Neil Douglas commanded the 79th Regiment for upwards of twenty-two years." P. 215.

It is a remarkable fact, and certainly one that is not very creditable to the intelligence or the good feeling of military officers in general, that of the many (214), who were examined before the Parliamentary Committee, appointed about ten years ago to report on military punishments, *two* only recommended any thing but physical penalties for the prevention of crime. In answer to the following query—*Are you enabled to suggest any means of restraining or eradicating the propensity to drunkenness, so prevalent among the soldiers, and confessedly the parent of the majority of military crimes?*—a great variety of penal enactments were advised; but no one suggested the schoolmaster's drill, but Sir George Arthur and the late Col. Oglander. The words of the latter breathe as much wisdom as benevolence: "The only effectual corrective of this, as of every other vice, is a sound and rational sense of religion. This is the only true foundation of moral discipline. The establishment of libraries, and the system of *adult* schools, would be useful in this view." P. 317.

It would be pleasing to follow out this more attractive part of our subject; but this is impossible; we must quit it for the present with this passing notice. What we have now to do with is the physical, rather than the mental, operation of Military punishments. And here it must be confessed, that the whole history of them is a sad and humbling record of cruelty, folly, and wickedness. Those, who may wish to become acquainted with details, will find much instructive matter in Dr. Marshall's narrative. Without alluding to the various modes in which capital punishment used to be inflicted, we shall do little more than merely enumerate some of the most approved of the secondary or minor punishments, many of which, be it remembered, have been in use until the last quarter of a century. These are the *strappado* or *estrapade*, hanging up by the thumbs, riding the wooden horse, lying neck and heels, picketing, running the gauntlet, cold burning, booting, the log, the stocks, the whirligig, slitting the nose, various sorts of mutilations, branding, imprisonment, &c. The *strappado* is thus described: "The delinquent is hoisted up by means of a rope fastened to the arms behind his back, and then suddenly dropped down with a jerk, by which process his shoulder-joints were sometimes dislocated. He was sometimes hoisted up, and again let fall two or three times."

"Picketing" was equally cruel and absurd:—

"The instrument here (Bombay, 1816) employed, consisted of a board, and a peg; the board, or block of wood, was about 12 inches long, eight broad and four thick. The peg, which tapered to a point about the size of a sixpence, was twelve inches in length, and inserted in the middle of the board. The punishment was inflicted as above described, the delinquent's right arm being fixed to a hook, and his left foot resting on the peg, while his left arm and right foot were tied together behind his back. Delinquents were sometimes kept on the peg for a period varying from ten to thirty minutes; and occasionally they fainted either while on the peg, or after they had been taken down." P. 154.

In former times, boring the tongue with a red-hot iron was a common penalty for blasphemy and swearing; the offending organ being made the suffering one, according to the strict principles of the *lex talionis*.

Flogging does not appear to have been used, as a regular form of punishment, in the British army for more than a century, if indeed so long; although manual correction had been doubtless well known at all times. During the rebellion in Scotland (1745) we read of the *cat* being repeatedly used to extort evidence as well as to punish the soldiers. Previously to this period, it would seem that rods were generally employed, and executioners hired for the purpose of inflicting the punishment. The *Fustuarium*, bastinado, or stick-beating, was a common punishment in the Roman armies.

For upwards of sixty years after the introduction of the *cat*, the mere arbitrary will of the commanding-officer appears to have been a sufficient warrant for its use upon a soldier's back; no court-martial was necessary. When our readers learn that it has been only since 1811, that captains of our ships of war have been required to forward to the Admiralty a regular report of the punishments inflicted by their command, they will cease to wonder at the extent of the enormities that were often perpetrated on board-ship. It was not much better on shore; save and except that no soldier could be flogged without (the form at least of) a trial. But this was often a mere mockery; a set of junior officers did as their commander bade them; and forthwith the soldier or soldiers were brought to the halberts. One of the most flagrant instances of the extreme lengths, to which unrestrained authority was apt to be carried in the infliction of punishment, is recorded in Sergeant Teesdale's *Letter addressed to the people of England*, 1835. In October, 1806, the 28th regiment embarked from the Cove of Cork for Germany. At Bremen, where they were stationed for some time, there was a parade every morning and afternoon, and scarcely a day passed without a flogging taking place for some frivolous offence or another. Sometimes 10, 15, and 25 men were flogged, one after the other.

"At one of the above flogging parades, when we had been nearly two hours witnessing the horrible scene of bloodshed, and when the hands and feet of every soldier in the regiment were benumbed from cold, and from remaining for such a length of time in one position; I say, at one of these parades, a brave old soldier, whose character was unimpeachable, happened to cough in the rank. He turned his head a little on one side to discharge the phlegm, and was instantly ordered into the centre of the square, stripped of his accoutrements, and placed in front of the halberts. He went through the mock form of a trial, by a drum-head court-martial. Major B—— swore he was unsteady in the ranks; and on the *ipse dixit* of that tyrant he was sentenced to receive fifty lashes. After the brave veteran was tied up he implored hard for mercy, adding, that he had been twenty years in the service, and was never till then brought to the halberts. The pale, worn, and dejected appearance of this man, from age and length of service, was in itself sufficient to excite compassion and sympathy, even had he been guilty of a crime; his appeal was useless, he had every lash of his sentence, weeping and crying bitterly during the infliction; and although he only received fifty lashes, he never looked up afterwards. It had wounded his best feelings; he was constantly in hospital, and but a little time elapsed before he was discharged." P. 171.

Lieutenant Shipp tells us that, when he was at Jersey in the year 1808, not a week passed over without some flogging exhibition. Several men in the 60th regiment, quartered there, were condemned to receive 1000 lashes for desertion. "This punishment," says he, "was rigidly inflicted, with the additional torture which must have resulted from the number of *five* being slowly counted between each lash; consequently the space of three hours and twenty minutes was occupied in inflicting the total punishment, as though 1000 lashes were not of themselves a sufficiently awful sentence without so cruel and unnecessary a prolongation of misery. Many of these poor creatures fainted several times, but having been restored to their senses by medicinal application, the moment they could move their heads the castigation was recommenced in all its rigour. Numbers of them were taken down and carried from the square in a state of utter insensibility. The spectacle, altogether, instead of operating as an example to others, created disgust and abhorrence in the breast of every soldier present who was worthy of the name of man."

Major Macnamara states that it is scarcely an exaggeration to say that, during the war, at least one half, if not three-fourths, of the soldiers of almost every regiment in the service had felt "the sting" of the lash. The mean number of lashes inflicted monthly in a regiment, then serving in India, was for some time 17,000! Strange to say, the reasonings of the West India Planters, in justification of their own barbarous conduct to their slaves, contributed—in a somewhat unexpected way, it must be acknowledged—to open men's eyes to the impolicy, not to say the iniquity, of such wholesale use of the lash in our military and naval services. The argument, as urged by "a native of Jamaica," who published a vindication of the treatment of the poor slaves, is so curious, and withal so cogent, that we must give it in his own words:

"In Europe, among free men, and by a court of free men, a seaman and a soldier are sometimes sentenced to receive 100 to 1000 lashes,—*men who have fought their battles and protected their liberty.* A master in the West Indies cannot, without answering to the laws for it, nor can a magistrate, by the settled laws of the country, give, or sentence a slave to receive, more at one infliction than forty lashes. Would not an idiot perceive on which side the *humanity lies?*" P. 183.

In 1812, by an order from the Horse Guards, no *regimental* court-martial could inflict more than 300 lashes at a time upon any offender.* Much credit is due to the late Duke of York for having taken this initiatory step in ameliorating the punishment of soldiers. Some officers of the old, unlimited flogging, school thought that this order would destroy the discipline of the army! Dr. Marshall amusingly tells us of one who swore that he would never comply with it; "for," said he, "my conscience would not allow me to award a sentence of 300 lashes when I felt convinced that a man deserved 600."

For many years after the above-named period, much, very much, remained to be done to make the penal code of the army at all consistent with any principles of humanity or justice. Among other enormities, the inhuman practice of inflicting the second—nay, sometimes the third, and even the fourth—part of a sentence upon a man, who had been declared by the surgeon incapable of receiving the whole amount of lashes at once, continued to prevail for many years afterwards; and this too, it deserves to be remarked, notwithstanding the recorded opinion of Mr. Manners Sutton, the Judge Advocate (1815), that the practice was clearly *illegal.* We read of one hideous instance, in which a man was sentenced to receive 1500 lashes for marauding. When brought to the halberts, he seized the drum-major's sword, and called upon his comrades to rescue him: they however did not interfere. He was forthwith flogged to the full extent of his sentence; subsequently he was tried for mutinous conduct in the square of the corps, found guilty, and shot! There was surely something shockingly barbarous in all this. The spirit, that dictated the double punishment, is revolting from its very vindictiveness. To torture a man first, and then put him to death, is it not horrible.

The practice in the Navy of flogging *through the fleet* is another disgraceful enormity, which should never be tolerated. Hear what a naval officer has said on the subject:

"I believe no man has ever been known to hold up his head after going

---

* It was during this year that, by an Act of the American Congress, flogging was entirely prohibited in the American army.

through the fleet. The heavy launch is fitted with a triangle, to which the wretch is tied, as if to a cross. It takes some hours to row (sometimes against wind and tide) through the fleet. The torture is, therefore, protracted till, to use a sailor's phrase, 'their very soul is cut out.' After this dreadful sentence they almost always die." P. 360.

In 1836, the award of a general court-martial was reduced to 200 lashes, of a district court-martial to 150, and of a regimental one to 100. Before that time, there was no limitation, as we have seen, to the extent of the sentence of a general court-martial: 1000 lashes were not unfrequently, and even 1500 were every now and then, ordered.

We have no intention to discuss the much vexed question as to the propriety of abolishing altogether corporal punishment by flogging; but we cannot pass over without notice some of the observations of Sir Charles Napier, recorded in his Treatise on Military Law, 1837; as they bear upon the duties of army surgeons, as well as upon the medical iniquity, if we may use the expression, of this mode of bodily chastisement. Among the objections which he urges against flogging, he very justly remarks that "it is torture of very unequal infliction;" varying, on the one hand, ac-according to the strength or temper of the drummers employed, not to mention the will of the commanding officer or drum-major; and, on the other, according to the different powers of endurance in different men, whether this difference may depend on original constitution or on the existing state of their health at the time. The following passage, in which Dr. Marshall comments on two of the other objections of the General, contains so much interesting information that we gladly submit it entire to our readers' perusal. The fifth objection by Sir Charles is worded thus:

"Because the state of a man's health, and the ability of a man to endure severe corporal infliction, cannot be always correctly ascertained."

"This is a most important objection, and one which has a particular reference to the duty of a medical officer. A medical officer of the greatest experience is unable to predict with certainty the effect of a severe, or even a moderate flogging, upon the future health of a man—either upon his general constitution, or locally upon his back. He may be attacked with fever, which may terminate in death; or inflammation and sloughing of the back may supervene, and be followed by a similar result. It is not to be expected that young or inexperienced medical officers should be acquainted with their duties on a punishment parade, more especially as no official instructions have been promulgated on that head. A medical officer ought to be informed in regard to what is required of him in the performance of this duty, and how he should execute it. Sir Charles Napier states that he knew of two soldiers who were flogged at Corfu, by sentence of a court-martial in 1819; both died, and neither had been punished with unusual severity. When an enquiry was made by Sir Thomas Maitland into the cause of their death, it was alleged that both of them had the '*malaria*' fever before they were punished, although neither they nor the medical officer who attended the punishment were aware of the circumstance. This allegation, or conjecture,—for it was only a conjecture,—served the purpose of shielding the Commanding Officer and surgeon from the consequences of any imputation of blame, in regard to the manner of carrying the sentence into effect. It is sufficiently well known that fever is frequently excited by injuries, such as the fracture of a bone, or a surgical operation, and it may also be caused by the contusions occasioned by a cat-o'-nine-tails. There is much truth in what Sir Charles Napier says on the subject. 'As to pulse,' says he 'it forms no criterion by which a medical officer may ascertain the state of a man's health; for what man's pulse would be regular when going to suffer, or when actually suffering torture?' I believe every possible care is in general now taken by medical officers, to ascertain the state of a man's health previous to his being flogged; but medical men cannot do what is impossible,—and it is impossible to say what may be the effect of corporal infliction with more certainty than to predict the consequence of a surgical operation. The most experienced surgeons can only guess; the result is, that when a man is tied up to be flogged, his life depends upon a guess, and that guess, perhaps, made by a young and inexperienced officer.

"Sixthly. Because the danger to life from flogging is greater in a tropical than in a temperate climate.

"The ratio of sickness in a tropical climate is more than double that of troops in a temperate climate; while the mean ratio of mortality in the former may be estimated at from four to six times that of the latter. Every cause of ill health, including the contusion excited by flogging, is of more importance in a warm than in a cold climate. Fever is more easily excited between the tropics than where the temperature is low.

"The fact is, (says Sir Charles Napier,) that the medical officers are placed in a most unfair and perilous position. The danger to which the life of the culprit and the *life of the surgeon* are exposed, appears to be a powerful objection to this punishment. As to making the surgeon responsible, it is unjust to do so: the law places a man *by force* in a certain position, and orders him to act according to the best of his judgment; he does so, and there is an end of the matter, whatever may be the consequences, unless it can be shewn that he was drunk or mad!" P. 225.

Our author has fully described the manner in which the various military punishments, including flogging, are carried into effect in our army. As a matter of course, we must refer the curious reader for details to the original. All that we shall do is to pick out a few passages, that may be supposed to have some interest in a medical point of view. When a man is to be flogged, he is, after stripping himself to the waist, tied to a machine termed a triangle, his hands being pulled up to the top of it and there secured; cords are passed round the upper part of the thighs, and the ancles. At other times, the delinquent is lashed to a gun-wheel or a tree. The amount of suffering from the punishment then inflicted may depend a good deal upon the manner in which the prisoner is secured. If the ligatures are too tight, the hands may become quite black and benumbed —the numbness continuing for several days—from the stoppage of the circulation; this arises partly from the man's hanging on, as it were, by the hands. If, again, they be too loose, he is liable to move and wriggle about from side to side, by which means the *cat* falls on unsuitable parts, as the ribs, the neck or even the face and eyes. During the last war, it was very common to flog upon the breech, and sometimes on the calves of the legs, when the back and breech were unsound from former inflictions. Flogging on the breech is said to be more painful than on the back, "probably in consequence of the greater sensibility of the extreme parts of the body."

"The first stroke of the cat," says Dr. Marshall, "occasions an instantaneous discoloration of the skin from effused blood, the back appearing as if it was thickly sprinkled with strong coffee, even before the second stroke. Sometimes the blood flows copiously by the time the first fifty or 100 lashes are inflicted; at other times, little or no blood appears when 200 lashes have been inflicted. During the first 150 or 200 lashes, a man commonly appears to suffer much, considerably more, indeed, than during the subsequent part of a punishment, however large it may be. The effused blood in the skin, or, perhaps, some disorganization of the nerves of sensation, seems to occasion a blunting of its sensibility, and thereby lessen the acuteness of the pain arising from the application of the cat. Left-handed drummers, whose cats are applied to a portion of sound skin, and drummers who have not been sufficiently drilled to flogging, spread the lashes unnecessarily, and excite an unusual degree of pain. Delinquents frequently call out to the drummer to strike higher, then lower, and sometimes alternately." P. 255.

An ex-drum boy, who subsequently rose to the rank of a commissioned officer, describes the result of his *experience* as a flogger, in the following terms:

"From the very first day I entered the service as drum-boy, and for eight years after, I can venture to assert, that, at the lowest calculation, it was my disgusting duty to flog men *at least three times a week.* From this painful task there was no possibility of shrinking, without the certainty of a rattan over my own shoulders by a Drum-Major, or of my being sent to the black-hole. When the infliction is ordered to commence, each drum-boy, in rotation, is ordered to strip, for the purpose of administering twenty-five lashes (slowly counted by the Drum-Major) with freedom and vigour. After a poor fellow had received about 100 lashes, the blood would pour down his back in streams, and fly about in all directions with every additional blow of the cat, so that by the time he had received 300, I have found my clothes all over blood from the knees to the crown of the head. Horrified at my disgusting appearance, I have, immediately after parade, run into the barrack-room, to escape from the observations of the soldiers, and to rid my clothes and person of my comrade's blood." P. 255.

Is not this truly revolting? but let us proceed in our narrative. Sir Charles Napier remarks that he has always observed that "when the skin is thoroughly cut up or flayed off, the great pain subsides. Men are frequently convulsed and screaming during the time they receive one lash to 300 lashes, and then they bear the remainder, even to 800 or 1000 lashes, without a groan: the drummers appear to be flogging a lump of dead raw flesh." Water is always at hand for the delinquent to drink, or for the purpose of sprinkling upon his face, should he become faint. Should the ends of the cords of the *cat* become entangled, they are disengaged from time to time; if clotted with blood, they are dipped in water. In former days, even this was not done; and the blood was allowed (ten being counted between each lash) to dry upon the cat, for the purpose of rendering the punishment more severe. It was then also, sometimes, the practice to steep the cat in brine before, as well as during, the infliction of the punishment: this brutality is now strictly prohibited. The *cat* we may here observe, is usually "made of a thick strong kind of whip-cord, (we quote the description of the ex-drum-boy), and on each lash, nine in number, and generally about two feet long, were tied *three* large knots, so that a poor wretch, who was doomed to receive 1000 lashes, had 27,000 knots cutting into his back, and men have declared to me, that the sensation experienced at each lash was as though the talons of a hawk were tearing the flesh off their bones."*

* The *cat* in the navy is a much more formidable instrument. The cord used is considerably larger: it is usually log-line. Moreover, a boatswain's mate

Will it be believed that the delinquent is actually made to pay for the use of the cat, with which he has been flogged? The charge is regularly entered in his monthly account, thus—" Drum-Major's charge, 6*d*." This is adding insult to injury with a vengeance!

Let us now briefly notice the duty of the medical officer in reference to flogging, in the army. Until within the last seven or eight years (1838), the surgeon did not necessarily know that a man was to be flogged until he saw him tied up; and not unfrequently he was ordered to attend at the punishment of men whom he had never seen before. Now-a-days no soldier can be punished until the surgeon has examined him, and certified that he is " in a good state of health, and fit to undergo corporal punishment or imprisonment, solitary or otherwise, and with or without hard labour." On this subject Dr. Marshall has the following very pertinent observations:—

" To certify that a soldier is fit for duty is sometimes attended with some difficulty; but to report a man to be able to undergo corporal punishment is a measure which requires still more careful consideration. In this country the duty in question is comparatively easy; but in tropical countries, where, in consequence of the prevalence of disease, every man is, on an average, two or three times in hospital in the course of twelve months, a regiment will always contain a considerable number of men who are not likely to endure the punishment of either flogging or imprisonment with impunity. The following example will illustrate the difficulty which sometimes occurs in the execution of this duty. A soldier belonging to—Regiment, serving in India, was sentenced to receive 100 lashes, a punishment which was commuted by the approving officer to imprisonment for a short period. The man had been carefully examined by a medical officer previously to trial, who emitted the required certificate of health and fitness to undergo corporal infliction. Within twenty-four hours after this man was confined, he was attacked with remittent fever, followed by *delirium tremens*, and narrowly escaped with his life. Had he received his sentence, 100 lashes, there is some reason for concluding that his career as a soldier would not have been long; and, perhaps, the medical officer might have been unjustly inculpated.

" During the great prevalence of endemic or epidemic disease, or when symptoms of scurvy or ill-conditioned sores appear among the men, it may be highly expedient for a medical officer to recommend that corporal punishments should not in any case be inflicted.

" In addition to the general health of a man, I would strongly recommend a medical officer invariably to pay much attention to the state of his mind. Military crimes, such as desertion and insubordination, are not unfrequently the result of idiotcy, weakness of intellect, or partial insanity, a circumstance of much importance in the administration of military discipline, and one which a medical officer would require carefully to investigate. Fictitious madness has no doubt been treated as real; but, what is of much more importance to my subject, real madness has escaped unobserved, or has been treated and punished as fictitious—a fearful mistake when the penalty was such a punishment as flogging." P. 269.

Dr. M. gives the particulars of a case, which it is very distressing to read. Medical men cannot be too sedulous in carefully and repeatedly scrutinising every particular, in a doubtful case.

---

can *lay on* with much greater force than a drummer. A dozen lashes on board-ship are supposed to be equal, in point of severity, to 50 given on parade.

It is altogether a very painful duty, and one too surely not devoid of degradation, for a medical man to determine the fitness or ability of a criminal to undergo a certain amount of torture. We have already seen what Sir C. Napier says upon this point. In the House of Commons, in 1834, we find that Sir Charles Grey expressed himself still more forcibly: "There are sentences of courts-martial which, if inflicted, would amount to loss of life; and I think when the punishment is to the extent which we sometimes hear of, it is *degrading rather to them who inflict it* than to the sufferer, and especially degrading to the noblest art to which human talent can attain—I mean the art of healing—when the attendance of a medical man is rendered necessary, *not to assuage pain and relieve suffering, but to ascertain the extreme limit of human endurance.*" Where is the medical officer that does not feel a sense of humiliation in such a responsibility being imposed upon him? Now, this responsibility is doubly onerous, when the crime, for which the man is tried, is that of "malingering," or feigning, or producing disease or deformity; because, not only is it mainly upon the evidence of the medical officer himself that the soldier must be usually convicted, but also because he is obliged to certify at the very same time that the prisoner is fit to undergo the punishment that is awarded. When we find so experienced an officer as our author admitting the difficulty, often very great, of distinguishing real from alleged suffering, disease or debility, and also of detecting some forms of mental alienation or imbecility, well does it become his younger brethren to consider their ways under the circumstances alluded to. His suggestion in the following passage indicates so much right feeling, as well as good sense, that it gives us great pleasure in making it more public.

"Instead of bringing an alleged malingerer at once before a court-martial, I think, with Dr. Cheyne, that a board, consisting of at least three medical officers of mature experience, would be found the better tribunal in the first instance, and upon their decision the ultimate measures should be grounded. It is easy to infer and to allege, that a soldier is fraudulently feigning a disability; but it is frequently very difficult to prove satisfactorily that he is not more or less affected with disease. No person should be convicted of the crime of malingering, unless it be very satisfactorily established by skilful and impartial testimony. A medical officer who has had the care of an alleged malingerer, and who has reported him to his commanding officer, may have so identified himself with the accusation, as to be somewhat biassed in his opinion of the case; and consequently a court-martial will be required to estimate the weight of his testimony, according to what they may deem its real value. I repeat my opinion, that no man should be punished for a delinquency so difficult to appreciate as feigning a disability, but upon the testimony of two competent and impartial witnesses." P. 270.

Dr. Marshall quotes a case, illustrative of these remarks, from the *Medico-Chirurgical Review*, No. 79, p. 75, in which a man, within not many hours of his death, was saved from an intended punishment by the prudent caution of the medical officer consulted.

There are still one or two points connected with the duties of the medical officer, in reference to corporal punishment by flogging, that deserve brief notice. He generally takes his station a few paces behind the sufferer; but should symptoms of fainting come on, he sometimes moves in front, that he may see his face. Every now and then, he feels the temporal pulse to guide him in forming his judgment. His business is to observe and watch the effects of the punishment; so that, on the one hand, the delinquent does not escape any part of it by simulating exhaustion or any other alarming symptom; and, on the other, that he is not permanently disabled for service, or have his life endangered by its severity. It is strange that, in the performance of this most responsible duty, the medical officer has no official instructions for the direction of his conduct: the mere usage of the service is his only guide. Should he observe any symptoms arise during the punishment, which, in his opinion, indicate the expediency of suspending its infliction, it is his duty to intimate the circumstance to the commanding-officer.

"But," our author goes on to remark, "as a Commanding Officer sometimes asks the surgeon whether the delinquent is not able to bear a greater number of lashes, he should invariably be prepared to give a suitable answer. A man may be able, in all probability, to endure a somewhat greater amount of punishment, without materially endangering his prospective fitness for the service; but it may be highly inexpedient in the opinion of a medical officer to sanction the infliction of a punishment to the utmost verge of safe endurance." P. 261.

Sir John Macdonald, in his evidence on Military Punishments, says that it is at the *peril* of a commanding officer to continue the punishment after the surgeon has interfered; but it does not appear that the former incurs any great risk, unless indeed the death of the sufferer ensue; for certain it is that commanding-officers have frequently refused to attend to the suggestions of the surgeon, without danger to themselves. Dr. M. tells us of one case, where the surgeon was actually put and kept in arrest for 10 days, in consequence of interference; when liberated, he could get no redress. It may be worthy of notice here, that in the celebrated case of Governor Wall—tried at the beginning of the present century for the murder (20 years before) of a soldier, who, by his commands, was flogged at Goree by negro-slaves, and died five days afterwards—the surgeon of the garrison was present during the greater part of the infliction, and did *not* recommend that the punishment should be suspended. Notwithstanding this, Wall was condemned and executed at the Old Bailey.

As to the responsibility of medical officers in respect of the consequences of excessive flogging, the following examples will show how serious it is, or, at least, can be interpreted to be.

On the 5th of February, 1824, at St. Vincent's in the West Indies, two men received 1000 lashes, each, for desertion. One died two days afterwards, apparently in consequence of collapse; the other, on the 14th, after a fit of ague: sloughing commenced on the 12th, and by the following day, the whole of the back and loins had become involved. The backs of the men were not much cut.

When the report was transmitted to the Inspector-general of hospitals at Barbadoes, he forthwith officially recommended that the surgeon, Mr. F———, (who, although he had been seven or eight years in the service, had never attended a punishment parade before) should be cashiered, on the ground, that, although 1000 lashes may be awarded by a general court-martial, it is never expected that the whole should be inflicted in a warm climate; and that to stand by and see 1000 lashes inflicted on men who had served long in a tropical country, evinced great want of feeling and judgment; it betrayed, he added, neglect or ignorance, or both, to a most discreditable degree.

The result was, that Mr. F. was ordered to England; and, in the following June, he was dismissed the service without court-martial, or his having any public opportunity of defending himself! Such treatment is surely quite irreconcileable with every principle of justice, when we read over and over again that punishment quite as severe, nay more so, has been repeatedly inflicted without any alarming consequences having ensued. Dr. Gordon Smith, in his *Forensic Medicine,* expressly says, "I have seen 1000 lashes received without complaint, and the back healed so rapidly that, in about ten days, the patient was dismissed cured." On the other hand, our author states it, as a remarkable fact, "that the fatal cases on record have, in most instances, been the result of very moderate inflictions, some even under 200 lashes." Dr. Hamilton (*Duties of a Regimental Surgeon considered,* 1794) mentions a case where, after 200 lashes, the sloughing became so deep and extensive that part of the back-bone and scapula was laid bare; the man was in hospital for seven months. Our author alludes to two similar cases of sloughing that occurred in his own experience: one man died; the other was never fit afterwards for duty, and required to be invalided. Need we add another word to shew the utter uncertainty of the eventual result of corporal punishment, according to individual circumstances which no medical man can ever fully or altogether be enabled to determine?*

A man under flogging may be seized, or feign to be seized, with fainting or convulsions at the very commencement of the punishment. The mere dread of it has been known to be followed by fatal consequences, although not a single lash had been given. Such a case is related by our author. It is sometimes by no means easy, as we have already stated, to distinguish real from feigned convulsive fits. Dr. Bell, in his work on *Diseases of Soldiers in the West Indies,* relates a case, where a soldier at first escaped punishment in consequence of such an attack, when he was brought to the halberds. On being afterwards convicted of a second offence, it was determined to go through with the punishment. Dr. Bell argued thus: "If the fits were feigned, the pain of the flogging would soon put an end to every exertion of artifice; and, if they were real, it appeared probable that severe pain, to which he had not been accustomed, and the operation of terror on his mind, at the time the fit was approaching, might prevent the attack, and, by breaking the habit, might prove a useful remedy." No ill effects resulted from the flogging; but Dr. Bell leaves us in doubt after all as to the real nature of the case; for he merely says, "whether the fits were real or feigned, impressing the mind with terror, produced the effect that was desired"—*i. e.* their non-recurrence.

Paralysis of one or of both arms has been known to be the result of flogging. Dr. M. has seen one such case; the man was discharged the service, in consequence of being disabled. In some cases, the constitution

---

* Persons of a sanguine temperament, with red or fair hair, and of a tall slender frame of body, usually suffer more than others. Fifty lashes have been known to disable a man of this sort for three months.

has been irrecoverably damaged by the extensive suppuration that has ensued. Sir H. Hardinge tells us that the Portuguese method of punishment, that of striking the culprit upon the back with the flat of the sword, is apt to be followed by spitting of blood, and other maladies. The same remark is made by Dr. Kirckhoff in reference to the Dutch army, in which the cane is generally used as the instrument of punishment.

The cruel consequences of second, and still more of third and fourth, instalments of punishment, may well be conceived, when we think of the thin and tender state of the newly-formed cuticle: the first few lashes usually bring the blood in streams down the man's back. We may remark that a commanding officer, it appears, has the power of remitting any part of the punishment awarded by a regimental court-martial, but not of a general one, when the sentence has been approved of by the sovereign or his representative. In the latter case, if the man should die before the entire punishment has been inflicted, (whether this be at once or at several times,) " it must be consummated upon his lifeless and mutilated carcase." So says Major James, in his *Regimental Companion,* 7th Edit. 1841 :—we trust that such is not the case.

The usual application to the back after flogging is a weak solution of sugar of lead. Whenever there is very great tumefaction of the parts, there is reason to apprehend the supervention of mortification. This is one of the most troublesome and dangerous consequences of the punishment: the putrid smell, more especially in hot climates, arising from the extensive gangrenous surface, is described as being sometimes almost unsupportable alike to the patient and attendants. We have already seen that no medical man can, with certainty, predict or foresee the degree of liability of the injured parts to fall into a state of sloughing ; much, very much, depends on the constitution of the patient, and other incidental circumstances.

When the sloughing and ulceration have been extensive, the cicatrised surface will sometimes remain so tender for a length of time as utterly to prevent the man wearing his cross-belts, far less carrying his knapsack. Several men have been dismissed the service in consequence.

It would seem, from Dr. Williamson's report, (Observations relative to the West India Islands, 1817), that, in the case of the poor negroes, the integuments of the back sometimes become nearly insensible from repeated thrashings.

" By frequently punishing offenders," says he, " the parts become insensible to that laceration which tears up the skin. When that barbarous consequence is arrived at, its infliction becomes a matter of indifference to the unfortunate negro ; and new sources of *torture* must be found out by which the commission of crime may be checked. It can scarcely be necessary to add, that such a condition of torpor in the parts to which punishment has been applied, can never be justified on any pretext; and I blush to reflect that white men should be the directors of such disgraceful deeds.—(*Observations relative to the West India Islands,* 1817.)

" Dr. Williamson had peculiar opportunities of acquiring information on this subject, having resided in a medical capacity during fourteen years upon different plantations in Jamaica.

" Allowing that few or none die, which (says Dr. Hamilton) I believe to be the fact, immediately from punishments moderately inflicted, I know, from experience in the service, that constitutions have been considerably impaired by them. We sometimes find the body melt away into a spectre of skin and bone, from the large suppurations that have followed ; nor were they ever afterwards, as long as I knew them, able to bear the same hardships as before; and they must from thence also be more incident, not only to contagious diseases, if they be in the way of them, but to other complaints to which fatigue or hardships of duty may expose them." P. 291.

Before closing our remarks, it may not be amiss to mention a professional epispastic which we find has been tried, and that too successfully, as a *succedaneum* for the coarse and barbarous one in common use in the army. Sir Charles Napier is the narrator.

"The Commanding Officer of one of the regiments in question, then stationed in Guernsey, where liquor is cheap, determined to try to put a stop to the crime of drunkenness on duty, by an appeal to the honourable feelings of soldiers, and at the same time to make drunkenness as unpleasant as possible, but without the lash. He gave out an order to say that he would not flog, but trust to the soldiers' self-respect for keeping sober on duty. Next day a man was drunk and confined. The Colonel, accompanied by the Surgeon, went to the guard-house, and felt the drunkard's pulse: he was declared to be in a fever. Nothing could be more true. He was therefore put into a blanket, and four soldiers bore him through the barracks, his comrades all laughing at the care taken of him; on reaching the hospital the patient was put to bed and *blistered* between the shoulders, fed on bread and water for a week, and then discharged cured. He was then brought on the parade, when the Commanding Officer congratulated him on his recovery from the fever, and sent him to join his company, when he was laughed at and jeered by his comrades during the space of a week. Many others underwent the same treatment; but the joke, though very amusing to the sober soldiers, soon began to be none to the drunkards. There was considerable pain and uneasiness—some bread, plenty of water; but no pitying comrades—no commiseration—no mercy. The experiment was completely successful. Not a man of that regiment was flogged in Guernsey from the time the men were treated with blisters: and after a fortnight there was no such thing as a man drunk for guard or parade. Now *this regiment had been in an infamous state.* 'Observe,' says Sir Charles, 'the consequence of having inefficient means. This same regiment was embarked for the Bermudas. There was at that period much drinking and much illness in these islands, rum being cheap and the blister-plaister scarce. There was no means of confinement, and the Lieutenant-Colonel, for want of efficient means, was obliged to use the lash, which punished without preventing drunkenness. Now the blister did prevent it in Guernsey. So much for inefficient means." P. 160.

Throughout the whole of his descriptions and remarks, Dr. Marshall displays a generous and enlightened feeling of condemnation of the barbarous and *unjust*—because *unequal*—punishment of flogging. There is something in it, too, positively degrading to the medical officers of the army and navy; from being messengers of mercy, they are made little better than aids of the executioner. Already has flogging been abolished among our native troops in India; nor does it exist, we believe, in the French, or in almost any of the European, armies. Without taking upon himself to say that it should be entirely given up in our public services, the general tone of our author's observations certainly tends to this result; and he strongly expresses his concurrence in the sentiments expressed by Dr. Hamilton, published upwards of fifty years ago in the following extract:

" *I wish, after all, the military laws knew no such thing as flogging, and that in place thereof some other mode of punishment could be devised less ignominious. On this head, however, I dare say nothing, it is out of my line of life, though I wish it, with all my soul, abolished, as an inhuman thing, more suiting the nature of savages than civilized and polished nations.*" P. 293.

And surely there is no occupation, in which medical men can more profitably fulfil their beneficent *mission,* than in using all their professional influence and authority to plead the cause of the suffering and oppressed against the heartless neglect or tyranical severity of irresponsible task-masters. Of recent years, the fruits of enlightened medical philanthropy have been gloriously displayed in the changes already effected in our prisons and lunatic asylums, &c. and in the improved general œconomic treatment of our pauper population. May the same good spirit continue to animate every member of the profession, in whatever sphere he may be placed! It is thus only that our calling may justly claim to itself the proud distinction, that has been assigned to it by the eloquent orator of antiquity, of being " an art almost divine."

